# Association between risk of dysphagia and signs suggestive of sarcopenia, nutritional status and frequency of oral hygiene in hospitalized elderly

**DOI:** 10.1590/2317-1782/20232022232en

**Published:** 2023-10-09

**Authors:** Ronivaldo Pinto Ferreira, Luana Marsicano Alves, Laura Davison Mangilli

**Affiliations:** 1 Programa de Pós-graduação em Ciências da Reabilitação, Faculdade de Ceilândia, Universidade de Brasília - UnB - Brasília (DF), Brasil.

**Keywords:** Dysphagia, Swallowing Disorders, Aging, Sarcopenia

## Abstract

**Purpose:**

To identify the risk of dysphagia and its association with signs suggestive of sarcopenia, nutritional status and frequency of oral hygiene in the hospitalized elderly.

**Methods:**

This is an analytical cross-sectional study with the participation of 52 elderly patients admitted to a medical clinic at a public hospital in the Federal District, Brazil. The Eating Assessment Tool, Strength, Assistance with walking, Rise from a chair, Climb stairs and Falls + Calf Circumference and the Mini Nutritional Assessment shortform were applied, in addition to the collection of sociodemographic data and health conditions.

**Results:**

Among the elderly participants, 30.8% were at risk of self-reported dysphagia. The factors associated with the risk of dysphagia were: signs suggestive of sarcopenia (p=0.04), nutritional status (p<0.001) and oral hygiene frequency (p=0.03).

**Conclusion:**

In the geriatric population of the present study, with the majority of the participants having tested positive for Covid-19, the risk of dysphagia was associated with signs suggestive of sarcopenia, nutritional status and frequency of oral hygiene.

## INTRODUCTION

Along with the geriatric syndromes, the natural aging process brings several morphological alterations that compromise human communication, including language, hearing, voice, and facial motricity. These impairments also affect the stomatognathic system, its structures - tongue, cheeks, jaw, lips, occlusal area, and palate, as well as its functions, such as suction, breathing, mastication, speech, and deglutition^(1)^.

This disability in the older population is generally manifested by difficulty in mastication or in starting the deglutition process, with the presence of cough, choking, heartburn, chest pain, nasal regurgitation during meals and a feeling of food stuck in the throat after eating. These effects make the dynamics of swallowing more vulnerable to disorders caused by minor health alterations, such as infections of the upper airways^(2)^.

These changes in the functionality of deglutition can be classified as dysphagia, an alteration defined as a “disrupted dietary habits”, with a condition that involves a perceived or actual difficulty to form or safely move a bolus from the oral cavity to the stomach^(3)^.

Over the past decade, such swallowing-related alterations have led to increasing awareness of the need to acknowledge oropharyngeal dysphagia as a geriatric syndrome that causes the following sequence of disorders: (1) Dehydration; (2) Anorexia → unwillingness to eat; (3) Weight loss → protein-energy undernutrition; (4) Sarcopenia → decline in function; (5) Aspiration: chemical or bacterial; (6) Decreased pleasure in eating/drinking; (7) Embarrassment in social situations; (8) Isolation → depression; (9) Caregiver stress; (10) Dysphoria; and (11) Death^(4)^.

The associated risk factors are unclear about the potential confusion factors or the mediators of dysphagia. Most studies are based on reverse causality and the preceding factors are not confirmed, nor is the deglutition alteration or the worsening health, such as sarcopenia, fragility and psychological state^(5)^.

Among these factors, sarcopenia is defined as a skeletal muscle disease with the main determinant being a low muscle strength that outweighs the function of low muscle mass^(6)^. Its tendency and increasing development in the older population, along with its consequences for oral function and deglutition, might result in oral fragility and indicate a poor health state^(7)^.

Such a decline in oral function and dysphagia can lead to undernutrition, aspiration pneumonia, asphyxiation and occasionally death. Malnutrition presents a high prevalence among the frail elderly, and dysphagia is independently associated with undernutrition. The decline in oral function can be an important predictor for the progression of malnutrition in older populations^(8)^.

Aspiration pneumonia, on the other hand, is one of the most critical complications of dysphagia, characterizing an infectious process caused by the bronchoaspiration of oropharyngeal secretions containing food residues and saliva with possible oral pathogens. Due to poor oral hygiene, saliva contaminated with a high amount of various species of bacteria can harbor microbes that, if colonized and aspirated, can result in bacterial pneumonia. As a result, the combined impacts of poor oral hygiene and dysphagia can increase the risk of aspiration pneumonia^(9)^.

Therefore, the older population presents a great risk of dysphagia due to the aging process. Such an alteration is frequent and belatedly detected, often associated with the senescence process, thus delaying investigations ^4,5^. The present study aimed to detect the risk of dysphagia and its correlation with signs suggestive of sarcopenia, nutritional status, and oral hygiene in the hospitalized elderly.

## METHODS

This is a cross-sectional study involving 52 older patients hospitalized in the patient care unit of a public hospital in the Federal District (DF), Brazil, between September and December 2021. The sample group was selected by convenience and composed of older individuals aged 60 years or older. We excluded from the population those individuals who presented severe cognitive impairments affecting perceptual ability, judgment and language, and/or those who had an amputated lower limb, were wearing orthopedic prostheses on their lower limbs, had edema or were on a suspended oral diet.

This study is part of the research project “Assessment of the risk of dysphagia in the hospitalized elderly and its relation to nutrition, sarcopenia, hydration and quality of life: a cross-sectional analytical and observational study”, approved by the Research Ethics Committee from the Ceilândia College of the University of Brasília (CEP/FCE - Faculdade de Ceilândia da Universidade de Brasília), decision number 3,749,828, and by the Research Ethics Committee from the Foundation for Teaching and Research in Health Sciences of the Federal District’s Health Department (CEP/FEPECS - Fundação de Ensino e Pesquisa em Ciências da Saúde, Secretaria de Saúde do Distrito Federal), decision number 3,820,960. All participants were instructed on the research objectives and data confidentiality and signed the Informed Consent Form.

Initially, the sociodemographic data and health information of the participants were researched, such as age, sex, education, race, color, pathologies, type of diet and dysgeusia. All data were collected with a structured survey by accessing the participant's medical record through the TrakCare® health information system of the Federal District’s Health Department and through questions answered by the participants at the time of anamnesis.

The frequency of oral hygiene was assessed by asking the participant how many times a day they performed it, with the following answer options: not at all; once; twice; three times; four or more times.

The Eating Assessment Tool^(10)^ (EAT-10) was subsequently applied, which is a practical tool for routine use in the care of older patients, being composed of a subjective and specific questionnaire to assess the degree of dysphagia symptoms. Each question has a score from 0 (no problems) to 4 (severe problem) and a maximum score of 40 points, with no risk of dysphagia for cohort scores < 3 points, and risk of dysphagia for scores higher than or equal to 3. The instrument starts with the question: “How much of a problem are these situations for you? Mark the best number for your case”, continuing with the following statements: my swallowing problem makes me lose weight; my swallowing problem keeps me from eating out; I have to force myself to drink liquids; I have to force myself to swallow food (solid); I have to force myself to swallow medicine; it hurts to swallow; my swallowing problem takes away my pleasure in eating; I get food stuck in my throat; I cough when I eat and swallowing makes me stressed. After the participant had responded, the scores of the corresponding answers in each item were added up and a result greater than or equal to three was an indicator of alteration.

The EAT-10 questionnaire application was followed by the SARC-F (Strength, Assistance with walking, Rise from a chair, Climb stairs and Falls) + Calf Circumference (CC) to detect the risk of sarcopenia. The ensuing questions were asked: How much difficulty do you have to lift or carry 5.0kg? How much difficulty do you have to walk across a room? How much difficulty do you have to get up from a chair or bed? How much difficulty do you have to climb 10 steps of a staircase? How many times have you fallen in the past year? The following answers were possible and quantifiable: none = 0, some = 1, a lot or cannot do it = 2, and for the question: how many times have you fallen in the past year? The measurable values of the answers were none = 0; 1-3 falls = 1; 4 or more falls = 2^(11)^.

Next, the anthropometric measurement of the Calf Circumference was collected. The assessment was performed with an inelastic tape measure on the most protruding part of the right leg, with the participant's leg bent to a 90-degree angle with the knee. To avoid the error bias inherent to CC measurements, this procedure was performed by a single anthropometrist (the main researcher). The assessment was conducted in bed for bedridden participants and sitting in a chair for ambulatory patients. The CC score was based on the EWGSOP2 ^7^ recommendation (2018): 0 points for women with >33 cm, 10 points for ≤33 cm; 0 points for men with >34 cm, and 10 points for ≤34 cm.

The SARC-F + CC questionnaire application was followed by the sum of the answer scores (0 - 20 points) and the participant was thusly classified: absence of signs suggestive of sarcopenia (0 - 10 points) and presence of signs suggestive of sarcopenia (11 - 20 points).

The risk of undernutrition was assessed by the Mini Nutritional Assessment Short-Form (revised MNA®-SF). The Brazilian Consensus on Nutrition and Dysphagia recommends that nurses apply the short version of this instrument to ensure the identification of at-risk older patients, as well as a possible referral for a nutritional assessment in cases of scores lower than or equal to 12^(12)^. One of the advantages of this version is the exclusion of redundant items that required special training, addressing the patient’s subjectivity and memory or generating too many blanks or “I don’t know” answers^(13)^.

The revised MNA®-SF is a screening composed of five questions with answer options corresponding to points (varying between 0 and 3) in the final sum. It assesses lower food intake, weight loss, mobility, psychological stress or acute illness, neuropsychological problems and Body Mass Index (BMI). In the end, the scores of the answers were added up to reach the final screening score: 12-14 points: regular nutritional status; 8-11 points: at risk of undernutrition, and 0-7 points: malnourished^(12,13)^.

The results were analyzed and interpreted on Microsoft Excel 2018 and the Statistical Package of Social Sciences (SPSS), version 19.0. The Chi-squared test was applied to detect whether there was an association between the EAT-10 variable classification and the qualitative variables. All analyses adopted a 95% confidence interval.

## RESULTS

The sample included 52 participants aged on average 73 (±8.3) years old. The average length of hospital stay at the day of the data collection was 5.5 (±3.86) days (Table 1). The sex of the participants was balanced, while the predominant race/color was white, followed by brown. Most of the patients were married and had an incomplete elementary education (Table 2).

**Table 1:** Mean age and hospitalization period in hospitalized older patients. Brasília, Federal District, 2022

**Variables**	**Mean**	**Standard Deviation**	**Minimum**	**Maximum**
Age (years)	73.17	8.38	61.00	94.00
Hospitalization period (days) on the day of the survey	5.50	3.86	1.00	15.00

Source: Research data, 2022

**Table 2:** Sociodemographic, clinical and functional characterization of hospitalized older patients. Brasília, Federal District, 2022

**Variable**	**Classification**	**N = 52**	**%**
Sex	Female	28	53.85%
	Male	24	46.15%
Race/Color	Yellow	1	1.92%
	White	32	61.54%
	Brown	18	34.62%
	Black	1	1.92%
Marital status	Married	21	40.38%
	Divorced	5	9.62%
	Single	5	9.62%
	Stable Union	5	9.62%
	Widower	16	30.77%
Education	Illiterate	7	13.46%
	Comp H.S.	8	15.38%
	Inc H.S.	1	1.92%
	Comp E.S.	6	11.54%
	Inc E.S.	26	50.00%
	Comp H.E.	4	7.70%
Reason for hospitalization	Surgical	12	23.08%
	Respiratory	40	76.92%
Covid-19	No	13	25.00%
	Yes	39	75.00%
Systemic arterial hypertension	No	18	34.62%
	Yes	34	65.38%
Diabetes mellitus	No	30	57.69%
	Yes	22	42.31%
Other comorbidities	No	18	34.62%
	Yes	34	65.38%
Complaints	Choking	5	9.62%
	Lack of appetite	1	1.92%
	Cough	13	25.00%
	No complaints	33	63.46%
Dentition	Permanent dentition	2	3.85%
	Edentulism	4	7.69%
	Partial prosthesis	12	23.08%
	Total prosthesis	34	65.38%
Type of diet	Bland/pasty	6	11.54%
	Bland	28	53.85%
	Enteral/pasty	3	5.77%
	Liquid	2	3.85%
	Pasty	13	25.00%
Feeding methods	Oral	49	94.23%
	Nasoenteric/oral probe	3	5.77%
Taste	Absent	2	3.85%
	Reduced	18	34.62%
	Normal	30	57.69%
	Complaint about tastes	2	3.85%
Frequency of oral hygiene	1	8	15.38%
	2	20	38.46%
	3	6	11.54%
	More than 3	18	34.62%
Smoking/alcoholism	Alcoholism	6	11.54%
	Smoking	1	1.92%
	Ex. alcoholic	5	9.62%
	Ex. smoker	11	21.15%
	Ex.smoker/Ex.alcoholic	11	21.15%
	Never smoked or consumed alcohol	18	34.62%

Caption: Comp H.S. = Complete High School; Inc H.S. = Incomplete High School; Comp E.S. = Complete Elementary School; Inc E.S. = Incomplete Elementary School; Comp H.E. = Complete Higher Education. Source: Research data, 2022

Respiratory problems were the main reason for hospitalization, with 39 (75.00%) of the older patients infected by the coronavirus (Covid-19).

Diabetes Mellitus (DM) and Systemic Arterial Hypertension (SAH) were found in most of the participants. Beside DM and SAH, 65.38% of the sample group presented other comorbidities. Most of the participants were on a mostly orally administered bland diet. Even though cough was an associated symptom, most of the patients denied such complaints. The data related to oral health showed that a large number of the participants used total denture and performed oral hygiene twice a day. As to smoking and alcoholism, most of the patients reported never having smoked or consumed alcohol, followed by those who had smoked and consumed alcohol but lost the habit.

Of the 52 participants, 30.8% were at self-reported risk of dysphagia according to the EAT-10, and 69.2% did not present such risk.

The data on Table 3 indicates that signs suggestive of sarcopenia were present in most (75.00%) of the 16 patients who presented the risk of dysphagia, while the 20 (55.60%) individuals who presented no symptoms of dysphagia showed no signs suggestive of sarcopenia. Therefore, there is a correlation between the EAT-10 and the SARC-F+CC classifications.

**Table 3:** Absolute and Relative Frequency of the EAT-10 Classification according to the SARC-F+CC classification in hospitalized older patients. Brasília, Federal District, 2022

**EAT-10 Classification**	**SARC-F+CC CLASSIFICATION**		**Total**	**Pearson’s chi-squared (p-value)**
	**No signs of sarcopenia**	**Signs suggestive of sarcopenia**		
Risk of dysphagia	4 (25.00%)	12 (75.00%)	16 (100.00%)	0.04
No risk of dysphagia	20 (55.60%)	16 (44.40%)	36 (100.00%)	
Total	24 (46.20%)	28 (53.80%)	52 (100.00%)	

Source: Research data, 2022

Table 4 shows that 11 out of the 16 participants with a risk of dysphagia were at risk of undernutrition; however, only 13 out of the 36 patients with no risk of dysphagia presented a regular nutritional status with significant statistical results.

**Table 4:** Absolute and Relative Frequency of the EAT-10 Classification according to the revised MNA®-SF classification in hospitalized older patients. Brasília, Federal District, 2022

**EAT-10 Classification**	**MNA CLASSIFICATION**			**Total**
	**Regular nutritional status**	**At the risk of undernutrition**	**Malnourished**	
Risk of dysphagia	1 (6.25%)	11 (68.75%)	4 (25.00%)	16 (100.00%)
No risk of dysphagia	13 (36.11%)	22 (61.11%)	1 (2.78%)	36 (100.00%)
Total	14 (26.92%)	33 (63.46%)	5 (9.62%)	52 (100.00%)

Pearson’s chi-squared p-value = <0.001. Source: Research data, 2022

According to Table 5, the EAT-10 classification is associated with the participant’s frequency of oral hygiene.

**Table 5:** Frequency of oral hygiene according to the EAT-10 classification in hospitalized older patients. Brasília, Federal District, 2022

**Frequency**	**Risk of dysphagia**		**No risk of dysphagia**		**Total**	
	**N**	**%**	**N**	**%**	**N**	**%**
1	4	25.0%	4	11.1%	8	15.4%
2	8	50.0%	12	33.3%	20	38.5%
3	3	18.7%	3	8.4%	6	11.5%
4 or more	1	6.3%	17	47.2%	18	34.6%
Total	16	100.0%	36	100.0%	52	100.0%

Pearson’s Chi-squared p-value = 0.03. Source: Research data, 2022

Tables 3, 4, and 5 and the variable EAT-10 classification depend on the variables regarding the SARC-F+CC classification, the revised MNA®-SF and the oral hygiene frequency, since the *p*-value for the test of these variables reached <0.05. Hence, was accepted the statistical hypothesis that the variables are dependent.

As statistically demonstrated, there is a correlation between the risk of dysphagia (EAT-10) and the variables of the SARCF+CC, the revised MNA®-SF and the oral hygiene frequency. Consequently, the odds ratio of individuals presenting risk of dysphagia (EAT-10) when exposed to alterations in these instruments (Table 6).

**Table 6:** Odds Ratio

**Variable**	**Exposition**	**Odds Ratio for the Risk of dysphagia EAT-10**	**95% CI**
Revised MNA®-SF	Undernutrition	52.00	2.616-1033.8
SARCF+CC	Signs suggestive of sarcopenia	3.75	1.013-13.88
Oral hygiene	≤3 times	13.42	1.59-112.64

Source: Research data, 2022

## DISCUSSION

Despite being balanced, the sample group composition revealed that most of the hospitalized elderly in the patient care unit between September and December 2021 were female, aged on average 73 years (minimum of 61 and maximum of 94 years), white, married and with incomplete elementary education. These findings corroborate a study^(14)^ carried out in 2016, whose participants were mostly female (67.1%) with a mean age of 71 years (minimum of 60 and maximum of 102 years).

The clinical data collected indicated respiratory reasons for hospitalization (76.92%) associated with covid-19 (75.00%). The literature reports that this number is within the cases related to the second pandemic wave, with a small reduction of cases in younger patients (until 49 years) and an increase in the older patients (≥60 years) among white individuals^(15)^. It is worth highlighting that the present study was not targeted to associating the risk of dysphagia with the covid-19 infection; however, that was the epidemiological scenario found.

As to the comorbidities, SAH and DM appeared, respectively, in 65.38% and 42.31% of the sample group, while other associated pathologies also occurred in 65.38%. Another study^(16)^ has also detected a greater occurrence of SAH among participants, describing it as present in 60.5% of the dysphagia patients. This proportion of individuals with SAH and DM found in the study indicates the demand for a greater vigilance and monitoring in order to prevent disabilities and their consequences in the quality of life of these individuals, as well as in the health system.

The hospitalized older patients at risk of dysphagia presented an association (*p*<0.05) with signs suggestive of sarcopenia. Among the 52 (100.0%) individuals in the sample, 12 (23.07%) presented the risk of dysphagia with symptoms of sarcopenia according to the SARC+F+CC. The risk of sarcopenia among elderly patients with a risk of dysphagia (16-100.0%) is even higher, encompassing 12 individuals (75.0%).

A study^(17)^ involving hospitalized patients also found similar results, with 38.8% of the participants with potential dysphagia also presenting sarcopenia. Conversely, another study^(18)^ pointed out the simultaneous occurrence of dysphagia and sarcopenia in 69.5% of hospitalized elderly, thus differing from our results. This discrepancy might be explained by the variation in the applied instruments and the health condition of the older individuals at the moment of the research.

Nevertheless, this number is considerably higher when older patients with signs suggestive of sarcopenia are analyzed separately, reaching 53.80% of the total sample. This population with no risk of dysphagia but presenting signs suggestive of sarcopenia should also be followed up and monitored throughout hospitalization to avoid further complications, since studies^(6,7,19)^ have demonstrated that sarcopenia is a significant predictor of mortality in the elderly. Therefore, it is important to diagnose and treat sarcopenia in order to reduce the associated mortality rates.

As above mentioned, 75.0% of the older individuals were hospitalized during the research period due to a Covid-19 infection. A study from 2020^(20)^ described that the coronavirus 2 (SARS-CoV-2) is a neurotropic virus that can cause peripheral nerve disease. Glossopharyngeal and vagal neuropathy, which are among the neurological manifestations of Covid-19, can lead to dysphagia.

In addition to this potential neurological manifestation, researchers have identified that the inflammatory process of the Covid-19, combined with undernutrition and low mobility during hospitalization, might predetermine a secondary sarcopenia and sarcopenic dysphagia. Nonetheless, only a few published studies have investigated the dysphagia in non-intubated individuals with Covid-19^(21)^.

Such epidemiological conditions of the Covid-19 found in 75.0% of the participants might have represented a bias since, as reported in other studies^(20,21)^, the SARS-CoV-2 infection is a risk factor for impaired swallowing.

Table 6 indicates that the hospitalized older patients exposed to signs suggestive of sarcopenia present 3.75 times more chances of being at risk of dysphagia according to the EAT-10, when compared to those who did not present such symptoms. Thereby, being exposed to the risk (signs suggestive of sarcopenia) increases the occurrence of a risk of dysphagia outcome.

Such an exposition to sarcopenia symptoms may result in sarcopenic dysphagia^(22)^, with lower muscle mass reducing the bolus propulsion strength, thus generating post-swallowing oropharyngeal residue retention. After the detection of the sarcopenia risk, it is important that the medical team follow up, namely, the speech-language pathologist must assess the tongue pressure strength, the nutrition team must evaluate the nutrient intake, and the nursing staff must monitor the food acceptability as well as the dietary supply, as prevention and rehabilitation measures.

The medical team should bear in mind that nutritional status is a relevant component for preserving the well-being and health of older individuals, especially upon hospitalization. An inadequate nutrition favors the onset of several diseases in this population, being a predisposing factor to fragility syndrome, sarcopenia and longer hospitalization periods^(23)^.

In this context, the present study found an association (p< 0.05) between the risk of dysphagia and the nutritional status of the hospitalized elderly. Among the 16 (100.0%) individuals at risk of dysphagia, 11 (68.75%) were at risk of undernutrition, 4 (25.00%) were malnourished and only 1 (6.25%) presented a regular nutritional status. As to the nutritional alterations of older patients with a risk of dysphagia, 93.75% presented an unfavorable nutritional status, probably requiring or having required a care plan and bedside monitoring.

Researchers have conducted a study^(24)^ with 49 older patients in a university hospital in Brasília and found similar results. Elderly individuals at risk of dysphagia and undernutrition represented 51.0% of the sample group, while malnourished patients at risk of dysphagia corresponded to 20.0%.

Meanwhile, another study^(25)^ investigated the correlation between the deglutition function and the nutritional status in older patients in Japan, finding that malnutrition occurred in 60.5% of the 38 (100.0%) patients at risk of dysphagia according to the EAT-10. The authors explained such a high occurrence by comparing the nutritional status and dysphagia characteristics while including the elderly at risk of malnutrition and malnourished in the same undernutrition classification.

After using a classification similar to that of these authors^(25)^, the present study also found a larger number of older individuals at risk of dysphagia and malnutrition (93.75%). Therefore, considering this classification related to a high number of hospitalized patients at risk of dysphagia associated with the nutritional status alteration, the findings herein indicate the need of tracking and monitoring the altered swallowing function and nutritional risk by the multi-professional team.

The present study carried out such a comparison including older individuals of the community due to the fact that the number of hospitalization days of the participants in the moment of research was considerably lower (5.5 ±3.86) than other studies^(26)^, and the risks could have been present before hospitalization. It is worth mentioning that this study was not aimed at pointing out the total hospitalization time, but the hospitalization time at the unit in the moment of the instruments’ application.

Results have indicated that such an association might be explained by the fact that dysphagia directly damages the ability to eat and drink, thus reducing the food intake of energy, water and other nutrients, resulting in malnutrition and dehydration. In older individuals, food and liquid ingestion are generally already reduced due to age-related alterations, in addition to social, emotional and health problems. Since undernutrition is associated with a loss of mass and muscle function, also affecting the mastication and deglutition muscles, dysphagia is a strong alteration of functionality and might onset the process of health risk in the elderly^(27)^.

However, by analyzing only the hospitalized older patients at risk of malnutrition and dehydration, it was found 63.46% and 9.62%, respectively, amounting to 73.08% of the altered sample, according to the revised MNA®-SF, which is a considerably high amount that requires tracking and monitoring by the medical team.

As depicted in Table 6, the hospitalized elderly exposed to undernutrition have 52 times more chances of presenting the risk of dysphagia according to the EAT-10, when compared to those who presented regular nutritional status. In other words, being exposed to the risk of malnutrition increases the occurrence of a risk of dysphagia outcome.

The Brazilian National Survey on Hospital Malnutrition^(28)^ (IBRANUTRE) assessed the nutritional status and prevalence of undernutrition in four thousand hospitalized individuals, in addition to evaluating the knowledge regarding the nutritional status and use of nutritional therapy by the medical teams. Malnutrition was present in 48.1% of the hospitalized individuals and severe malnutrition occurred in 12.5%, nevertheless, there is little medical knowledge on undernutrition and nutritional therapy is underprescribed. Malnutrition presented a correlation with the primary diagnosis on admission, age (60 years), presence of cancer or infection, and a longer hospital stay (*p* < 0.05).

Some studies^(23,29)^ have demonstrated that these alterations and correlations might be initially involved with anorexia of aging. As age advances, food ingestion decreases, muscle mass is reduced and body fat mass increases. The causes of anorexia of aging include: decreased sense of smell and taste with lesser association; alterations in the gastric fundus compliance due to nitric oxide deficiency; decreased antral stretch with greater association with postprandial anorexia; as well as gastroparesis in response to large meals.

As already mentioned, most of these hospitalized elderly had no attendants throughout hospitalization due to the Covid-19, which might have interfered with food ingestion and resulted in the undernutrition risk.

The frequency of oral hygiene by the older individuals studied showed to be correlated with the risk of dysphagia. Most of them (53.0%) reported performing less than three oral hygiene procedures a day, which might be associated both to the lack of habit, since most of them used dental prostheses, and/or a lack of encouragement, assistance as well as instruction by the medical team throughout hospitalization.

The hospitalized elderly exposed to the risk of performing oral hygiene up to three times a day presented 13.42 times more chances of dysphagia risk according to the EAT-10, when compared to those who performed oral hygiene more than three times a day (Table 6). Thus, the exposition to this risk (≤3 oral hygiene procedures/day) increases the occurrence of a risk of dysphagia outcome.

It is paramount to preserve good oral health in hospitalized geriatric patients, since it is strongly associated with nutritional ingestion, lower risk of respiratory and cardiovascular diseases and better quality of life. Oral diseases caused by plaque were identified as a major risk for the patient’s ability to eat, communicate, and socialize^(30,31)^.

Such a low frequency of oral hygiene among the hospitalized elderly can be explained by the reports indicating that most of the time there is a lack of attendants or caretakers, which could have encouraged or helped the patients with their hygiene. A research study^(32)^ addressed the issue of how many frail and medically compromised older individuals are still able to perform oral and prosthesis hygiene by themselves, and how many of them need help to perform this activity. Oral hygiene and cleaning of dentures require a certain level of manual dexterity, visual acuity, procedural and cognitive skills, along with a sufficient mobility of the shoulder as well as elbow joints.

Further studies addressing the adaptation process of older patients experiencing such harmful alterations to functional and satisfactory swallowing should be conducted to establish a better technical and supportive understanding of the risk detection, for a better quality of life and recovery of these individuals. Further research should also investigate the matter of financial costs, suggesting instruments that are easy and low cost to apply, promoting a more controlled monitoring of the risk of dysphagia, saving resources without harming the quality of the services.

It is worth highlighting that the EAT-10, the SARC-F+CC and the revised MNA®-SF instruments were applied to detect the risk of dysphagia, signs suggestive of sarcopenia and risk of undernutrition, respectively. In the case of a positive result, the health care professionals conducting the evaluation must issue a referral of the patient to a speech-language pathologist and/or nutritionist, and/or physician for diagnostic assessment and therapy intervention.

The non-statistical findings herein show that all individuals with a risk of dysphagia according to the EAT-10 (16/30.8%) were hospitalized due to respiratory problems resulting from a Covid-19 infection. Thus, the present study indicates the need for research addressing the risk of dysphagia in older patients hospitalized due to Covid-19, in order to find whether this health condition is associated with sarcopenia, nutritional status and oral hygiene frequency.

The challenge for the future is to disseminate and broaden the recognition of dysphagia as an important geriatric syndrome, in addition to showing its impact on the older population to healthcare professionals, especially during hospitalization. Further instruments with high sensitivity and specificity should be developed for a faster and easier application.

## CONCLUSION

The elderly population studied herein, mostly tested positive for Covid-19, presented a risk of dysphagia associated with signs suggestive of sarcopenia, nutritional status and oral hygiene frequency.
