# Supplementary material for: Association between risk of dysphagia and signs suggestive of sarcopenia, nutritional status and frequency of oral hygiene in hospitalized elderly
Source: Codas. 2023 Oct 9;36(1):e20220232. doi: 10.1590/2317-1782/20232022232en (PMC10715578; doi:10.1590/2317-1782/20232022232en)
Supplement: Translation

# Translation: Associação entre risco de disfagia e sinais sugestivos de sarcopenia, estado nutricional e frequência de higiene oral em idosos hospitalizados

**DOI:** 10.1590/2317-1782/20232022232pt

**Authors:** Ronivaldo Pinto Ferreira, Luana Marsicano Alves, Laura Davison Mangilli

**Affiliations:** 1 Programa de Pós-graduação em Ciências da Reabilitação, Faculdade de Ceilândia, Universidade de Brasília - UnB - Brasília (DF), Brasil.

**Keywords:** Disfagia, Transtornos da Deglutição, Envelhecimento, Sarcopenia

## Abstract

**Objetivo:**

Identificar o risco de disfagia e sua associação com os sinais sugestivos de sarcopenia, estado nutricional e frequência da higiene oral em idosos hospitalizados.

**Método:**

Trata-se de um estudo transversal analítico com participação de 52 idosos internados em clínica médica de um hospital público no Distrito Federal. Foram aplicados os instrumentos Eating Assessment Tool, Strength, Assistance with walking, Rise from a chair, Climb stairs and Falls + Circunferência da Panturrilha e o Mini Nutritional Assessment shortform além de coleta de dados sociodemográficos e de condições de saúde.

**Resultados:**

Dos idosos participantes 30,8% apresentaram risco de disfagia autorrelatada. Os fatores associados ao risco de disfagia foram sinais sugestivos sarcopenia (p=0,04), estado nutricional (p<0,001) e frequência da higiene oral (p=0,03).

**Conclusão:**

Na população geriátrica deste estudo, em sua maioria com Covid-19, o risco de disfagia esteve associado aos sinais sugestivos de sarcopenia, estado nutricional e frequência da higiene oral.

## INTRODUÇÃO

O processo natural de envelhecimento junto com as síndromes geriátricas, traz diversas alterações morfológicas que comprometem a comunicação humana, entre elas: alterações de linguagem, audição, voz e motricidade facial. Esse comprometimento afeta o sistema estomatognático: suas estruturas - língua, bochechas, mandíbula, lábios, área oclusal e palato, e suas funções - sucção, respiração, mastigação, fala e deglutição^(1)^.

Geralmente, esse comprometimento na população idosa se manifesta pela dificuldade em mastigar ou iniciar o processo de deglutição, com a presença de tosse, engasgos, pirose, dor torácica e regurgitação nasal durante as refeições e sensação de alimento parado na garganta após as refeições. Estes efeitos tornam a dinâmica da deglutição mais vulnerável a distúrbios causados por pequenas alterações de saúde, como infecções de vias aéreas superiores^(2)^.

Essas modificações na funcionalidade da deglutição poderão ser classificadas como disfagia, uma alteração definida como uma “alimentação perturbada” com condição que envolve dificuldade percebida ou real em formar ou mover um bolo alimentar com segurança da cavidade oral até o estomago^(3)^.

Com essas alterações relacionadas à deglutição, na última década, tem havido uma crescente conscientização de que a disfagia orofaríngea deve ser reconhecida como uma síndrome geriátrica. Isso porque ela ocasiona uma sequência de distúrbios como descrita a seguir: (1) Desidratação (2) Anorexia → relutância em se alimentar (3) Perda de peso → desnutrição energética proteica (4) Sarcopenia → diminuição da função (5) Aspiração: química ou bacteriana (6) Diminuição do prazer de comer / beber (7) Constrangimento em situações sociais (8) Isolamento → depressão (9) Estresse do cuidador (10) Disforia (11) Morte^(4)^.

Os fatores de risco associados não esclarecem quanto aos potenciais fatores de confusão e aos mediadores da disfagia. Os estudos, em sua maioria, estão sujeitos a causalidade-reversa e não se confirma o que vem antes, a alteração na deglutição ou o agravo à saúde, como por exemplo a sarcopenia, fragilidade e o estado psicológico^(5)^.

Dentre esses fatores a sarcopenia é definida como uma doença do músculo esquelético, com a baixa força muscular superando o papel da baixa massa muscular como principal determinante^(6)^. Devido a sua tendência e desenvolvimento crescente na população idosa, suas consequências na função oral e na deglutição, podem resultar em fragilidade oral e indicar um mau estado de saúde^(7)^.

Essa diminuição da função oral e a disfagia podem levar à desnutrição, pneumonia por aspiração, asfixia e, às vezes, morte. A desnutrição apresenta alta prevalência entre os idosos frágeis, e o risco de disfagia está independentemente associado à desnutrição. A diminuição da função oral pode ser um importante preditor da progressão da desnutrição em populações idosas^(8)^.

Já a pneumonia aspirativa, uma das complicações mais críticas da disfagia, é um processo infeccioso provocado pela broncoaspiração de secreções orofaríngeas contendo resíduos alimentares e saliva com possíveis patógenos orais. Devido à má higiene oral, a saliva contaminada com uma quantidade elevada de várias espécies de bactérias pode abrigar micróbios que, se colonizados e aspirados, podem resultar em pneumonia bacteriana. Como resultado, os impactos combinados de má higiene oral e disfagia podem aumentar o risco de pneumonia por aspiração^(9)^.

Dessa forma, a população idosa apresenta grandes riscos para disfagia, devido aos efeitos do processo de envelhecimento. Essa alteração é frequente e tardiamente identificada, sendo associada, muitas vezes, a este processo de senescência e, assim, postergando as investigações^4,5^. Este estudo tem o objetivo de identificar o risco de disfagia e sua associação com os sinais sugestivos de sarcopenia, estado nutricional e frequência da higiene oral em idosos hospitalizados.

## MÉTODO

Trata-se de um estudo transversal realizado com 52 idosos internados em unidade de enfermaria, clínica médica, de um hospital público no Distrito Federal (DF) durante o período entre setembro e dezembro de 2021. A população amostral, selecionada por conveniência, foi constituída por idosos, com idade igual ou superior a 60 anos. Foram excluídos idosos que apresentaram alterações cognitivas graves que afetavam a capacidade perceptiva, de discernimento e de linguagem e/ou que possuíam algum membro inferior amputado, estivesse em uso de próteses ortopédicas em membros inferiores, com edema ou que estavam com dieta via oral suspensa.

O presente estudo é derivado do projeto de pesquisa “Avaliação do risco de disfagia em idosos hospitalizados e sua relação com a nutrição, sarcopenia, hidratação e qualidade de vida: um estudo transversal analítico e observacional”, aprovado pelo Comitê de Ética e Pesquisa da Faculdade de Ceilândia da Universidade de Brasília (CEP/FCE), conforme parecer número 3.749.828 e pelo Comitê de Ética e Pesquisa da Fundação de Ensino e Pesquisa em Ciências da Saúde da Secretaria de Saúde do Distrito Federal (CEP/FEPECS), conforme parecer número 3.820.960. Todos os participantes foram informados sobre os objetivos da pesquisa e a confidencialidade dos dados e assinaram o Termo de Consentimento Livre e Esclarecido.

Inicialmente, foi realizada a busca de dados sociodemográficos e de saúde dos participantes como exemplo a idade, sexo, escolaridade, raça, cor, patologias, tipo de dieta ofertada e alterações no paladar, que foram coletadas por meio de um levantamento estruturado acessando o prontuário do participante através do sistema *TrakCare*® de informação em saúde da Secretária de Saúde do Distrito Federal e através de perguntas respondidas pelo participante no momento da anamnese.

A frequência da higiene oral foi levantada perguntando ao participante quantas vezes ele realizava a higiene oral ao dia, tendo as seguintes opções de resposta: não realizo; uma vez; duas vezes; três vezes; quatro ou mais vezes.

Em seguida foi aplicado o *Eating Assessment Tool* ^(10)^ (EAT-10) que é um instrumento prático, para uso rotineiro no cuidado aos idosos; é um questionário subjetivo e específico para avaliar o grau dos sintomas da disfagia. Cada questão tem uma pontuação de 0 (sem problemas) a 4 (problema grave) sendo a pontuação máxima de 40 pontos e a notas de coorte < 3 pontos sem risco para disfagia e maior ou igual a 3 com risco para disfagia. O instrumento aborda o participante questionando “o quanto essas situações são um problema para você? Marque o melhor número para o seu caso”, e segue com os questionamentos: meu problema de engolir me faz perder peso; meu problema para engolir não me deixa comer fora de casa; preciso fazer força para beber líquidos; preciso fazer força para engolir comida (sólido); preciso fazer força para engolir remédios; dói para engolir; meu problema para engolir me tira o prazer de comer; fico com comida presa/ entalada na garganta; eu tusso quando como e engolir me deixa estressado. Após o participante ter respondido, somaram-se os valores das respostas correspondentes em cada item onde um resultado maior ou igual a três foi indicador de alteração.

Após aplicação do EAT-10 foi iniciado o questionário SARC-F (*Strength, Assistance with walking, Rise from a chair, Climb stairs and Falls*) + Circunferência da Panturrilha (CP) para análise do risco de sarcopenia. Foram aplicadas as seguintes perguntas: Quanta dificuldade o senhor (a) tem para levantar ou carregar 5,0kg? Quanta dificuldade o senhor (a) tem para andar por um quarto? Quanta dificuldade o senhor (a) tem para levantar de uma cadeira ou cama? Quanta dificuldade o senhor (a) tem para subir 10 degraus de escada? Quantas vezes o senhor (a) caiu no último ano? As respostas possíveis e quantificáveis foram: nenhuma = 0, alguma = 1, muita ou não consegue = 2, e para a pergunta: quantas vezes o senhor (a) caiu no último ano? Os valores mensuráveis das respostas foram: nenhuma = 0; 1-3 quedas = 1; 4 ou mais quedas = 2^(11)^.

Em seguida foi realizada a medida antropométrica da circunferência da panturrilha. A medida foi realizada na perna direita, com uma fita métrica inelástica, na sua parte mais protuberante, com o participante com a perna dobrada formando um ângulo de 90 graus com o joelho e para evitar o viés de erro inerente à CP, esse procedimento foi realizado por um único antropometrista (o pesquisador principal). Em participantes acamados a aferição foi realizada no leito e em deambulantes sentados em uma cadeira. A pontuação para CP seguiu o recomendado pelo EWGSOP2^7^ (2018): mulheres >33cm 0 pontos; ≤33 cm 10 pontos; homens >34cm 0 pontos e ≤34cm 10 pontos.

Após aplicação do questionário SARC-F + CP fez se o somatório dos valores das respostas (0 - 20 pontos) e o participante foi classificado em: ausência de sinais sugestivos de sarcopenia (0 - 10 pontos) e sugestivo de sarcopenia (11 - 20 pontos)

O risco de desnutrição foi avaliado pela aplicação da *Mini Nutritional Assessment Short-Form* (MNA-SF *revised*®). O consenso brasileiro de nutrição e disfagia recomenda a aplicação, pelo enfermeiro, dessa versão reduzida, garantindo a identificação dos idosos em risco e a possibilidade de indicação quando o escore for menor ou igual a 12 para avaliação específica com nutricionista^(12)^. Podemos citar como benefícios dessa versão a exclusão de itens redundantes, que necessitavam de treinamento especial, que envolviam subjetividade e memória do paciente ou que produzissem muitas respostas em branco ou “não sei”^(13)^.

O MNA-SF *revised*® é um questionário de triagem composto por 5 perguntas com opções de respostas correspondentes a pontos (podem variar de 0 a 3), para somatória no final da avaliação. Aborda diminuição da ingesta alimentar, perda de peso, mobilidade, estresse psicológico ou doença aguda, problemas neuropsicológicos e Índice de Massa Corpórea (IMC). Ao final foi realizada a somatória dos números correspondentes às respostas para obter o escore final da triagem: 12-14 pontos: estado nutricional normal; 8-11 pontos: sob risco de desnutrição e 0-7 pontos: desnutrido^(12,13)^.

Para a análise e interpretação dos resultados foram utilizados os programas Microsoft Excel 2018 e o “*Statistical Package of Social Sciences*” (SPSS) versão 19.0. A fim de averiguar se existe uma associação entre a variável classificação EAT- 10 e as variáveis qualitativas foi utilizado o teste de Qui-Quadrado. O nível de confiança adotado neste estudo foi equivalente a 95%.

## RESULTADOS

A amostra foi composta por 52 participantes com idade média de 73 (±8,3) anos. O tempo de internação, tendo a relação com o dia da coleta de dados, apresentou uma média de 5,5 (±3,86) dias (Tabela 1). O sexo dos participantes encontra-se na amostra de forma equilibrada, predominância da cor/raça branca, seguido dos pardos. A maior parte eram casados e, em relação ao nível de escolaridade, possuíam o ensino fundamental incompleto (Tabela 2).

**Tabela 1:** Média de idade e tempo de internação em idosos hospitalizados. Brasília, DF, 2022

**Variáveis**	**Média**	**Desvio Padrão**	**Mínimo**	**Máximo**
Idade (Anos)	73,17	8,38	61,00	94,00
Tempo de Internação (Dias) no dia da pesquisa	5,50	3,86	1,00	15,00

Fonte: Dados da pesquisa, 2022

**Tabela 2:** Caracterização sociodemográfica, clínica e funcional em idosos hospitalizados. Brasília, DF, 2022

**Variável**	**Classificação**	**N = 52**	**%**
Gênero	Feminino	28	53,85%
	Masculino	24	46,15%
Raça/Cor	Amarela	1	1,92%
	Branca	32	61,54%
	Parda	18	34,62%
	Preta	1	1,92%
Estado civil	Casado	21	40,38%
	Divorciado	5	9,62%
	Solteiro	5	9,62%
	Uni.Estável	5	9,62%
	Viúvo	16	30,77%
Escolaridade	Analfabeto	7	13,46%
	E.M. Comp	8	15,38%
	E.M.Inc	1	1,92%
	E.Fun.Comp	6	11,54%
	E.Fun.Inc	26	50,00%
	E.S. Comp	4	7,70%
Motivo internação	Cirúrgico	12	23,08%
	Respiratório	40	76,92%
Covid-19	Não	13	25,00%
	Sim	39	75,00%
Hipertensão arterial sistêmica	Não	18	34,62%
	Sim	34	65,38%
Diabetes mellitus	Não	30	57,69%
	Sim	22	42,31%
Outras comorbidades	Não	18	34,62%
	Sim	34	65,38%
Queixas	Engasgos	5	9,62%
	Falta apetite	1	1,92%
	Tosse	13	25,00%
	Sem queixas	33	63,46%
Dentição	Dentição permanente	2	3,85%
	Dentulismo	4	7,69%
	Prótese parcial	12	23,08%
	Prótese total	34	65,38%
Tipo de dieta	Branda/pastosa	6	11,54%
	Branda	28	53,85%
	Enteral/pastosa	3	5,77%
	Líquida	2	3,85%
	Pastosa	13	25,00%
Via da dieta	Oral	49	94,23%
	Sonda nasoenterica/oral	3	5,77%
Paladar	Ausente	2	3,85%
	Diminuído	18	34,62%
	Normal	30	57,69%
	Queixa de gostos	2	3,85%
Frequência da higiene oral	1	8	15,38%
	2	20	38,46%
	3	6	11,54%
	Mais de 3	18	34,62%
Tabagismo/etilismo	Etilismo	6	11,54%
	Tabagismo	1	1,92%
	Ex. etilista	5	9,62%
	Ex. tabagista	11	21,15%
	Ex.tabagista/Ex.etilista	11	21,15%
	Nunca fumaram ou consumiram álcool	18	34,62%

Legenda: E.M. Comp = Ensino Médio Completo; E.M.Inc = Ensino Médico Incompleto; E.FUN.Comp = Ensino Fundamental Completo; E.FUN.Inc = Ensino Fundamental Incompleto; E.S. Comp = Ensino Superior Completo. Fonte: Dados da pesquisa, 2022

A questão respiratória foi o motivo de internação principal, estando infectados pelo Coronavírus (Covid-19) 39 (75,00%) dos idosos.

O Diabetes Mellitus (DM) e a Hipertensão Arterial Sistêmica (HAS) foram encontrados em grande parte dos participantes. Além do DM e HAS, 65,38% apresentaram outras comorbidades. No que tange a dieta, a maioria dos participantes estavam com dieta do tipo branda, com predominância da via de administração oral. A queixa relacionada a disfagia mais afirmada foi a tosse, mas a maioria dos participantes negaram queixas relacionadas. Os dados relacionados à saúde bucal mostraram que uma grande parte estavam em uso de prótese dentária total e realizavam a higiene oral duas vezes ao dia. Em relação ao tabagismo e etilismo ocorreu a predominância de afirmações de nunca terem fumado ou consumido bebida alcoólica, seguido por aqueles que já fizeram uso de cigarros e álcool, mas não possuem mais o hábito.

Dos 52 participantes 30,8% apresentaram risco de disfagia autorrelatada pelo EAT-10 e 69,2% não apresentaram esse risco.

Os dados da Tabela 3 nos mostra que dos 16 participantes que apresentaram risco de disfagia os sinais sugestivos de sarcopenia estavam presentes na maioria (75,00%), e 20 (55,60%) dos que não apresentaram sinais sugestivos de disfagia também não apresentaram sinais sugestivos de sarcopenia. Há associação entre a Classificação do EAT-10 com a classificação da SARC-F+CP.

**Tabela 3:** Frequência Absoluta e Relativa da Classificação EAT-10 segundo a classificação SARC-F+CP em idosos hospitalizados. Brasília, DF, 2022

**Classificação EAT-10**	**CLASSIFICAÇÃO SARC-F+CP**		**Total**	**Qui-quadrado de Pearson (p-valor)**
	**Sem sinais de sarcopenia**	**Sugestivo de sarcopenia**		
Risco de disfagia	4 (25,00%)	12 (75,00%)	16 (100,00%)	0,04
Sem risco de disfagia	20 (55,60%)	16 (44,40%)	36 (100,00%)	
Total	24 (46,20%)	28 (53,80%)	52 (100,00%)	

Fonte: Dados da pesquisa, 2022

A Tabela 4 nos apresenta que dos 16 participantes com risco de disfagia, 11 estavam sob o risco de desnutrição, mas que dos 36 que não apresentaram risco de disfagia somente 13 apresentavam estado nutricional normal, com resultados estatísticos significantes.

**Tabela 4:** Frequência Absoluta e Relativa da Classificação EAT-10 segundo a classificação MNA -SF *revised*® em idosos hospitalizados. Brasília, DF, 2022

**Classificação EAT-10**	**CLASSIFICAÇÃO MNA**			**Total**
	**Estado nutricional normal**	**Sob risco de desnutrição**	**Desnutrido**	
Risco de disfagia	1 (6,25%)	11 (68,75%)	4 (25,00%)	16 (100,00%)
Sem risco de disfagia	13 (36,11%)	22 (61,11%)	1 (2,78%)	36 (100,00%)
Total	14 (26,92%)	33 (63,46%)	5 (9,62%)	52 (100,00%)

Qui-quadrado de Pearson p-valor = <0,001. Fonte: Dados da pesquisa, 2022

Conforme a Tabela 5, a classificação EAT-10 está associada com a frequência com que o participante realiza a sua higiene oral.

**Tabela 5:** Frequência da higiene oral segundo a classificação EAT-10 em idosos hospitalizados. Brasília, DF, 2022

**Frequência**	**Risco de disfagia**		**Sem risco de disfagia**		**Total**	
	**N**	**%**	**N**	**%**	**N**	**%**
1	4	25,0%	4	11,1%	8	15,4%
2	8	50,0%	12	33,3%	20	38,5%
3	3	18,7%	3	8,4%	6	11,5%
4 ou mais	1	6,3%	17	47,2%	18	34,6%
Total	16	100,0%	36	100,0%	52	100,0%

Qui- quadrado de Pearson p-valor = 0,03. Fonte: Dados da pesquisa, 2022

Nas tabelas 3, 4 e 5 a variável classificação EAT-10 é dependente das variáveis classificação SARC-F+CP, classificação MNA-SF *revised*® e frequência da higiene oral pois o *p*-valor apresentado para o teste dessas variáveis foram <0,05, logo aceitamos a hipótese estatística de que as variáveis são dependentes.

Como demonstrado estatisticamente há uma associação do risco de disfagia (EAT-10) com as variáveis do SARCF+CP, MNA-SF *revised*® e frequência da higiene oral. Desta forma, realizou-se a razão de chances (*odds ratio*) dos indivíduos apresentarem risco de disfagia (EAT-10) estando expostos as alterações nesses instrumentos (Tabela 6).

**Tabela 6:** Razão de chances

**Variável**	**Exposição**	***Odds Ratio* Risco de disfagia EAT-10**	**IC 95%**
MNA -SF *revised*®	Desnutrição	52,00	2,616-1033,8
SARCF+CP	Sugestivo de sarcopenia	3,75	1,013-13,88
Higiene oral	≤3 vezes	13,42	1,59-112,64

Fonte: Dados da pesquisa, 2022

## DISCUSSÃO

A composição da amostra revelou que mesmo estando balanceada, a maioria dos idosos hospitalizados na clínica médica entre setembro e dezembro de 2021 pertenciam ao sexo feminino, média de idade de 73 anos (mínimo de 61 anos e o máximo de 94 anos), afirmaram ser da raça/cor branca, casados e possuíam ensino fundamental incompleto. Esses achados vão ao encontro com outro estudo^(14)^ realizado em 2016 onde a maioria eram do sexo feminino (67.1%) com média de idade de 71 anos (mínimo de 60 anos e máximo de 102 anos).

Os dados clínicos levantados apontaram que o motivo das internações foi respiratório (76,92%) tendo ligação com o Covid-19 (75,00%). A literatura traz que esse número está dentro dos casos relacionados à segunda onda da pandemia, onde houve pequena redução proporcional de casos nas faixas etárias mais jovens (até 49 anos de idade) e aumento nas faixas mais idosas (≥60 anos) entre indivíduos de raça/cor branca^(15)^. Lembramos que não foi objetivo do estudo associar o risco de disfagia com a infecção pelo Covid-19, sendo esta a realidade epidemiológica encontrada.

Em relação as comorbidades, a HAS e o DM apresentaram respectivamente em 65,38% e 42,31% da amostra, estando também outras patologias associadas em 65,38%. Essa maior presença de HAS entre os participantes, também foi identificada em outro estudo^(16)^, que apontou essa característica em 60,5% dos pacientes portadores de disfagia. Essa proporção de participantes com HAS e DM apresentada no estudo, demonstra a necessidade de uma maior vigilância e monitoramento para prevenção de incapacidades e de suas consequências na qualidade de vida desses indivíduos e no sistema de saúde.

Os idosos hospitalizados com risco de disfagia apresentaram associação (*p*<0,05) com sinais sugestivos de sarcopenia. Entre os 52 (100,0%) indivíduos da amostra, 12 (23,07%) apresentaram risco de disfagia com sinais sugestivos de sarcopenia pelo SARC+F+CP. Quando se observa esse risco de sarcopenia dentro dos idosos com risco de disfagia (16-100,0%) esse número apresenta-se mais alto 12 (75,0%).

Um estudo^(17)^ com indivíduos hospitalizados também encontrou resultados semelhantes, 38,8% dos participantes com provável disfagia apresentaram também sarcopenia. Outro estudo^(18)^ demonstrou simultaneamente disfagia e sarcopenia em 69,5% dos idosos hospitalizados, divergindo dos resultados desse estudo. Essas diferenças podem ser explicadas pela variação dos instrumentos aplicados e quadro de saúde dos idosos no momento da pesquisa.

Mas quando é analisado separadamente só os idosos com sinais sugestivos de sarcopenia o número aumenta consideravelmente para 53,80% da amostra total. Essa população sem risco de disfagia, mas com sinais sugestivos de sarcopenia também deverá ser acompanhada e monitorada durante a hospitalização com o intuito de evitar futuras complicações, pois estudos^(6,7,19)^ vem demonstrando que a sarcopenia é um preditor significativo de mortalidade por todas as causas entre os idosos. Dessa forma, é importante diagnosticar e tratar a sarcopenia para diminuir as taxas de mortalidade por essa causa.

Como mencionado anteriormente 75,0% dos idosos estavam hospitalizados por motivo de infecção pelo Covid-19 durante a pesquisa. Em um estudo de 2020^(20)^ os autores descreveram que o coronavírus 2 (SARS-CoV-2) é um vírus neurotrópico que pode provocar doenças nos nervos periféricos. A neuropatia glossofaríngea e vagal, que estão entre as manifestações neurológicas da Covid-19, pode levar a disfagia.

Além dessa possível manifestação neurológica, pesquisadores identificaram que o processo inflamatório da Covid-19 combinado com a desnutrição e baixa mobilidade durante a hospitalização, pode predeterminar o indivíduo à sarcopenia secundária e disfagia sarcopênica, no entanto, há poucos estudos publicados sobre disfagia em indivíduos não intubados com Covid-19^(21)^.

Esse quadro epidemiológico da Covid-19 encontrado em 75,0% dos participantes pode ter sido um possível motivo de viés, já que como demonstrado em outros estudos^(20,21)^, a infecção pelo SARS-CoV-2 é um fator de risco para alterações na deglutição.

Na Tabela 6 podemos observar que idosos hospitalizados expostos aos sinais sugestivos de sarcopenia apresentam 3,75 vezes mais chances de apresentarem risco de disfagia pelo EAT-10 em comparação aos que não apresentam esses sinais. Sendo assim, a exposição ao risco (sinais sugestivos de sarcopenia) aumenta a ocorrência do desfecho de risco de disfagia.

Essa exposição aos sinais sugestivos de sarcopenia, pode acarretar na disfagia sarcopênica^(22)^, onde redução de massa muscular diminuiu as forças de propulsão do bolus alimentar resultando em resíduo orofaríngeo pós-deglutição. É importante que após essa identificação de risco para sarcopenia a equipe de saúde, em especial o fonoaudiólogo, avalie a força de pressão da língua, a equipe de nutrição acompanhe o aporte nutricional e a enfermagem o processo de aceitação e oferta da dieta como prevenção e reabilitação do indivíduo.

A equipe de saúde assistencial, deverá ter em mente que o estado nutricional é um relevante componente para manter o bem-estar e a saúde de idosos, ainda mais se estes estiverem hospitalizados. Uma nutrição inapropriada para este público contribui para o aparecimento de várias doenças e é fator predisposto para a síndrome da fragilidade, sarcopenia e aumento do tempo de internação^(23)^.

Dessa forma, o estudo apresentou uma associação (p< 0,05) entre o risco de disfagia e o estado nutricional dos idosos hospitalizados. Dentre os 16 (100,0%) indivíduos com risco de disfagia, 11 (68,75%) estavam sob risco de desnutrição, 4 (25,00%) estavam desnutridos e somente 1 (6,25%) apresentou estado nutricional normal. Considerando as alterações nutricionais nesses idosos com risco de disfagia, temos que 93,75% apresentavam um estado nutricional desfavorável e que provavelmente necessitariam ou necessitaram de um plano de cuidados e monitoramento beira leito.

Pesquisadores realizaram um estudo^(24)^ com 49 idosos em um hospital universitário de Brasília e chegaram a resultados semelhantes. Idosos com risco de disfagia e risco de desnutrição representaram 51,0%, já os desnutridos e com risco de disfagia 20,0%.

Já outro estudo^(25)^ que investigou a associação entre a função da deglutição e o estado nutricional em idosos da comunidade no Japão, mostraram que entre os 38 (100,0%) indivíduos com risco de disfagia pelo EAT-10 a desnutrição esteve presente em 60,5%. Esse número mais alto é explicado porque os autores quando realizaram a comparação das características para o estado nutricional e disfagia incluiriam os idosos com risco de desnutrição e desnutridos na mesma classificação de desnutrição.

Quando realizamos uma classificação semelhante à realizada pelos autores^(25)^ temos um número maior de idosos com risco de disfagia e desnutrição (93,75%). Portanto, considerando essa classificação com alto número de indivíduos hospitalizados em risco de disfagia e alteração no estado nutricional, o presente estudo traz a necessidade de rastreamento e acompanhamento pela equipe multiprofissional nas alterações da deglutição e risco nutricional.

Foi feita essa comparação com idosos da comunidade por motivo de que os dias de internação dos participantes no momento da pesquisa era consideravelmente pequeno (5,5 ±3,86) em comparação a outros estudos^(26)^ e os riscos apresentados poderiam já estar presentes também na comunidade antes da hospitalização. Vale lembrar que não foi objetivo do estudo demonstrar o tempo total de internação e sim o tempo de internação na unidade no momento da aplicação dos instrumentos.

Resultados indicam que essa associação pode ser explicada pelo fato de que a disfagia prejudica diretamente a capacidade de comer e beber, reduz a ingestão alimentar de energia, água e outros nutrientes, resultando em desnutrição e desidratação. Em idosos, a ingestão de alimentos e líquidos geralmente já está reduzida devido a alterações relacionadas à idade e devido a problemas sociais, emocionais ou de saúde. Como a desnutrição é acompanhada de perda de massa e função muscular, afetando também os músculos mastigatórios e de deglutição, a disfagia é uma forte alteração de funcionalidade e pode desencadear processos de riscos à saúde de idosos^(27)^.

Mas quando se analisa somente os idosos hospitalizados com risco de desnutrição e desnutrição esse estudo apresentou respectivamente 63,46% e 9,62% totalizando 73,08% da amostra alterada segundo a MNA-SF *revised*®. É consideravelmente um número alto que requer rastreio e monitoramento por parte da equipe assistencial.

Conforme a Tabela 6, idosos hospitalizados expostos à desnutrição têm 52 vezes mais chances de apresentarem risco de disfagia pelo EAT-10 em comparação aos que apresentam estado nutricional normal. Sendo assim, a exposição ao risco (desnutrição) aumenta a ocorrência do desfecho de risco de disfagia.

Um Inquérito Nacional Brasileiro de Nutrição Hospitalar^(28)^ (IBRANUTRE) avaliou o estado nutricional e a prevalência de desnutrição em 4000 mil indivíduos hospitalizados, além do conhecimento do estado nutricional pelas equipes de saúde e o uso de terapia nutricional. Foi demonstrado que a desnutrição esteve presente em 48,1% dos hospitalizados e a desnutrição grave em 12,5%, já o conhecimento médico sobre desnutrição é baixo e a terapia nutricional é subprescrita. A desnutrição apresentou relação com o diagnóstico primário na admissão, idade (60 anos), presença de câncer ou infecção e maior tempo de internação (*p* < 0,05).

Há estudos^(23,29)^ demonstrando que essas alterações e relações podem estar envolvidas inicialmente com a anorexia do envelhecimento. Com o envelhecimento, há uma diminuição da ingestão alimentar, juntamente com um declínio da massa muscular e um aumento da massa gorda. Dentre as causas da anorexia do envelhecimento podemos citar: a diminuição do olfato e do paladar com menor relação; alterações na complacência do fundo do estômago devido à deficiência de óxido nítrico e diminuição do estiramento antral com maior relação na anorexia pós-prandial, assim como o retardo do esvaziamento gástrico em resposta a grandes refeições.

Como já mencionamos anteriormente, esses idosos hospitalizados estavam em sua maioria sem acompanhantes durante a hospitalização por motivo do Covid-19 e isso poderá ter interferido na ingesta alimentar e ter proporcionado um quadro de risco para desnutrição.

Já a quantidade de vezes que os idosos realizam a higiene oral apresentou associação com o risco de disfagia. A maioria dos idosos (53,0%) relataram menos de três procedimentos de higienização oral por dia. Isso pode estar ligado tanto a cultura de não ter a prática do hábito, visto que a maioria fazia uso de prótese dentária e/ou a falta de incentivo, ajuda e orientação por parte da equipe de saúde durante a hospitalização.

Idosos hospitalizados expostos ao risco de realizarem até três higienes orais ao dia apresentam 13,42 vezes mais chances de apresentarem risco de disfagia pelo EAT-10 em comparação aos que realizam mais de três higienes orais ao dia (Tabela 6). Sendo assim, a exposição ao risco (≤3 higienes orais/dia) aumenta a ocorrência do desfecho de risco de disfagia.

Durante a hospitalização a preservação de uma boa saúde bucal é importante para pacientes geriátricos, pois está fortemente relacionada à ingestão nutricional, diminuição do risco de doenças respiratórias e cardiovasculares e melhor qualidade de vida. As doenças bucais causadas pela placa bacteriana foram identificadas como um grande risco para a capacidade do paciente de comer, comunicar e socializar^(30,31)^.

Essa baixa frequência de higiene oral em idosos hospitalizados, apresentada no estudo, pode ser explicada pelos relatos que na maior parte do tempo eles não tinham acompanhantes ou cuidadores que poderiam incentivar ou ajudar na higienização. Uma pesquisa^(32)^ relatou que em idosos frágeis e medicamente comprometidos, coloca-se a questão de quais destes ainda são capazes de realizar uma higiene bucal e da prótese suficiente sozinhos ou que precisam de assistência para essa atividade, pois a higienização oral e a limpeza das próteses necessitam de um certo nível de destreza manual, acuidade visual, habilidades processuais, cognitivas e mobilidade suficiente das articulações do ombro e cotovelo.

Novos estudos, que abordam o processo de adaptação do idoso às alterações prejudiciais para uma deglutição funcional e satisfatória, deverão ser realizados para uma melhor compreensão técnica e assistencial na identificação de riscos, melhora da qualidade de vida e reabilitação destes. É preciso também estudos de custos financeiros, que demonstrem que instrumentos de fácil aplicação e baixo custo, poderão favorecer o rastreio de risco de disfagia com controle e racionalização de recursos empregados, sem que se perca a qualidade dos serviços prestados.

Lembramos que os instrumentos EAT-10, SARC-F+CP e a MNA-SF *revised*®, são utilizados respectivamente para a identificação de pessoas com risco de disfagia, sinais sugestivos de sarcopenia e risco de desnutrição. Se a identificação for positiva, o profissional identificador deverá realizar o encaminhamento para o fonoaudiólogo e/ou nutricionista e/ou médico para a avaliação diagnóstica e condutas terapêuticas.

Em nosso estudo, foi possível observar, não estatisticamente, que todos os indivíduos com risco de disfagia pelo EAT-10 (16/30,8%), tinham como motivo de internação a questão respiratória decorrente da infecção pelo Covid-19. Dessa forma, esse estudo apontou a necessidade de pesquisas envolvendo o risco de disfagia em idosos hospitalizados por Covid-19, demonstrando se há associação desse quadro de saúde com a sarcopenia, estado nutricional e frequência da higiene oral.

O desafio futuro é divulgar e aumentar o reconhecimento da disfagia como uma importante síndrome geriátrica e demonstrar aos profissionais de saúde seu impacto na saúde da população, em especial nos idosos durante uma hospitalização. É importante o desenvolvimento de mais instrumentos com alta sensibilidade e especificidade com rápida e fácil aplicação.

## CONCLUSÃO

Na população geriátrica deste estudo, em sua maioria com Covid-19, o risco de disfagia esteve associado aos sinais sugestivos de sarcopenia, estado nutricional e frequência da higiene oral.
